# Analysis of the Health Examination Service Process Using Service Blueprint: Focus on the Older Adult Patient in South Korea

**DOI:** 10.3390/healthcare11202709

**Published:** 2023-10-10

**Authors:** Donghee Kim, Jungeun Cho

**Affiliations:** Department of Business Administration, Kyungsung University, Busan 48434, Republic of Korea; hkiiim@ks.ac.kr

**Keywords:** health examination services, older adult patient, healthcare service, service process, service blueprint, health screening service

## Abstract

As the older adult population grows, the paradigm of aging is shifting from simply living longer to living longer while maintaining health. This shift has led to a transformation in healthcare from passive to proactive approaches, emphasizing disease prevention. Health examination services have seen significant growth as they transition from being solely diagnostic processes to crucial tools for disease prevention. This study focuses on the health examination service industry, particularly in the context of the older adult population, and aims to develop a service blueprint to identify challenges and solutions in utilizing these services. The research employs the service blueprint methodology to map out the health examination service process comprehensively. The distinction is made between customer interactions and internal procedures that are observable and those that are not. Through a comprehensive analysis of the service process, it is possible to identify potential instances of customer unhappiness. These instances primarily occur during the initial interaction between older clients and the service, as well as when they receive their examination findings. There are several factors that contribute to discontent among individuals, namely the insufficient comprehension of the needs of the aged by service providers and the provision of substandard facilities. The study suggests strategies to improve customer satisfaction, such as dedicated personnel for older adult assistance, specialized education for staff, and better facilities tailored for older adult patients. Furthermore, the research highlights the significance of effectively addressing both areas of critical discontent and elements that enhance satisfaction in the process of service design. This research provides a detailed analysis of health examination services for the older adult, highlighting opportunities for improvement through enhanced customer experiences and specialized services.

## 1. Introduction

The demographic composition of South Korea is experiencing a notable shift as the share of older adult people is on the rise. In the year 2020, the population of individuals aged 65 and above in South Korea amounted to 8.84 million, or approximately 16.7% of the overall population [1]. The projected growth rate of this figure is anticipated to reach 24.6% by the year 2040 [2]. As the older adult population grows in South Korea, the concept of living long is evolving to include not only “how long to live,” but also “how to live while remaining healthy”. Due to these changes, health care is shifting from passive to preventive and active concepts. The demand for services is increasing significantly as health examination services, which were previously considered only a process of disease diagnosis, are now recognized as an important means of disease prevention. Since the enactment of South Korea’s Labor Standards Act in 1953, which mandated regular health examinations for some employees in certain workplaces, the health examination service market has seen significant growth, with market sizes ranging from a minimum of 8 trillion KRW to a maximum of 18.5 trillion KRW annually [3]. The number of examination institutions has also witnessed substantial growth, increasing from a mere 1980 institutions in 1980 to a staggering 20,957 institutions as of 2016. When compared to the estimated size of the domestic pharmaceutical industry market at 21.7 trillion KRW, it becomes evident that this is a tremendously substantial market [4]. According to Korean national statistics, the number of individuals receiving general health check-ups provided by the National Health Insurance increased significantly from 11,419 individuals in 2012 to 16,953,007 individuals in 2021 [5]. Korea’s public regular health checkup program was introduced based on the Japanese system. In Japan, health checkups are conducted based on individual laws such as the Law on Ensuring Medical Care for Elderly People and the Labor Standards Act, with health insurance providers and employers taking the lead. In the United Kingdom, the government centrally manages the health checkup program, focusing on two main aspects: newborn screening and cancer screening. In the United States, health checkup services are primarily provided by private insurance companies, and there is no centralized standardized public program [3].

Health examination services are a type of preventive health care that aims to reduce disease-related damage through early detection and treatment. Health examination services are defined as medical examinations (e.g., counseling, scientific examination, pathological examination, and radiology tests) conducted by health examination institutions for the purpose of checking health conditions, and the prevention and early detection of diseases. With the increasing size of the older adult population and the upward trajectory of income levels in South Korea, there is a corresponding expansion in the significance of the health examination service industry and the associated demands placed upon it. According to the 2014 Korea Health Industry Development Institute, 65.3% of respondents indicated their willingness to use health care support services to promote and maintain health in the future [6].

Services are consumed concurrently with their production, and the engagement of customers in the production process directly influences the customer’s experience at service encounters, thereby exerting a direct impact on the assessment of service quality [7]. This is why service process management is deemed the foremost crucial element in quality management. Furthermore, due to the intangible nature of service processes, they are more challenging to manage compared to manufacturing processes. From this standpoint, recent perspectives have shifted from a focus on the technical or functional aspects of services to a greater emphasis on understanding the situations and environments customers find themselves in. Interacting with customers and creating customer value through these interactions are now regarded as more vital, contrasting with past viewpoints [8,9].

Health examination services offer a range of services that encompass not just sickness treatment and inspection, but also health promotion initiatives. Despite significant investments in technology pertaining to this matter, there is a lack of active analysis and consumption of services. There has been a lack of comprehensive investigation into the utilization of health examination services from the standpoint of older individuals, who are more prone to encountering challenges in communication, decision-making, and physical abilities as a result of sensory decline and age-related cognitive impairment. In recent years, there has been a growing interest in the study of customer experience at the service point. However, most of the research has focused on general customer experience in hospitals [10,11,12,13], and there have been few studies that have identified and analyzed service points based on the characteristics of older adult patients [14]. This is a significant gap in the literature, as older adult patients may have different needs and expectations than general patients. Further research is needed to identify and address the specific needs of older adult patients at the service point, in order to improve their customer experience and overall health outcomes.

The primary objective of this study is to address these knowledge gaps by formulating a comprehensive service plan. Specifically, our study aims to evaluate the present landscape and identify challenges linked to the utilization of health checkup services among older adults. By meticulously dissecting the resources and processes underpinning service provision, we endeavor to gain profound insights into potential obstacles and craft effective remedies for each stage of the process. This holistic approach promises to enhance the overall user experience, a matter of particular significance for older adults who confront distinctive impediments while endeavoring to access and navigate health examination services.

## 2. Research Design and Methodology

### 2.1. Service Blueprint

**Figure 1:**
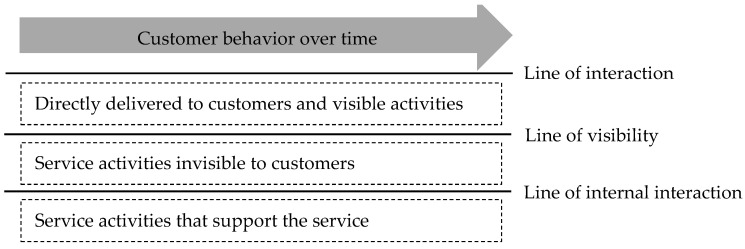
The concept of service blueprint.

Unlike visible products, managing intangible services and improving their quality necessitates an organic analysis of the processes required to deliver the services and the various facilities required to proceed [15]. This assists in determining whether customer demands are produced and communicated to customers efficiently. Although numerous techniques can be used for analyzing the process from service creation to consumption, Shostack’s [16] service blueprint, an analysis technique that schematically represents the delivery process, is the most widely used. A service blueprint is a flow chart-based design tool that defines a service and accurately identifies who is receiving the service, what each service is, and how it is delivered. It also applies to the blueprint by standardizing the customer’s process from start to finish. The service blueprint is made up of three lines: visibility, interaction, and internal interaction. A line of visibility distinguishes between actions in the service that are directly exposed to customers and those that are not. Meanwhile, a line of interaction classifies the behavior of service providers that provide services through direct contact with customers, and an internal line of interaction distinguishes between activities that support the service and others. Furthermore, the horizontal axis of the service blueprint represents both the customer’s and the service provider’s behavior, whereas the vertical axis distinguishes where the service is generated (Figure 1).

Following the clarification of the definition of the service to be analyzed, we must determine who should participate in the service’s schematic process. In most cases, service managers and experts with a thorough understanding of the service process should participate, and in some cases, customers should be included [17].

The service blueprint provides a quick view of the service organization’s complex business, enables managers to understand the entire process accurately, and provides information required for pre-verification when developing new services. It identifies failure points to prevent service failures and opportunities for service improvement [18].

Because of these benefits, the service blueprint has been applied to various service industries. Lee et al. [19] and Chung and Cho [20] used a service blueprint to analyze the airline’s in-flight service process, present customer complaints, and find ways to improve the service. Park [21] examined the outpatient service process in general hospitals and presented improvement measures in the form of a blueprint to improve efficiency.

### 2.2. Subjects and Duration of Study

The data used in this study were collected from four general hospitals in South Korea that operate health examination centers. A general hospital, as defined by the Korean Medical Service Act, must have a minimum of 100 beds and, depending on the bed capacity, should offer a specific number of medical specialties, each with dedicated specialists [22]. Basic information about the hospitals that were the subjects of this research has been summarized and presented in Table 1. The research was conducted over approximately one month, from September to October 2017, using methods such as direct observation and in-depth interviews.

Initially, a total of 10 experts, comprising health examination service center personnel from four hospitals and professionals in service quality, geriatric nursing, social welfare, and hospital administration, were organized into four teams. These teams were tasked with visiting the hospitals to conduct an analysis of the flow within the health examination services. Following that, a total of seven advisory sessions were held to delineate and define the specific activities that would be undertaken inside the service. Visits were conducted to prominent national organizations specializing in health examination services to assess any supplementary factors, leading to the formulation of an initial framework for health examination services.

In the next phase, a group meeting was organized, involving 28 individuals, comprising managers and practitioners from 20 health examination centers, to review and make additions or modifications to the service blueprint. Discussions were also conducted pertaining to customer concerns and instances of service deficiencies that may arise at different encounters during the service delivery procedure.

## 3. Results

### 3.1. Health Examination Services Blueprint

**Figure 2:**
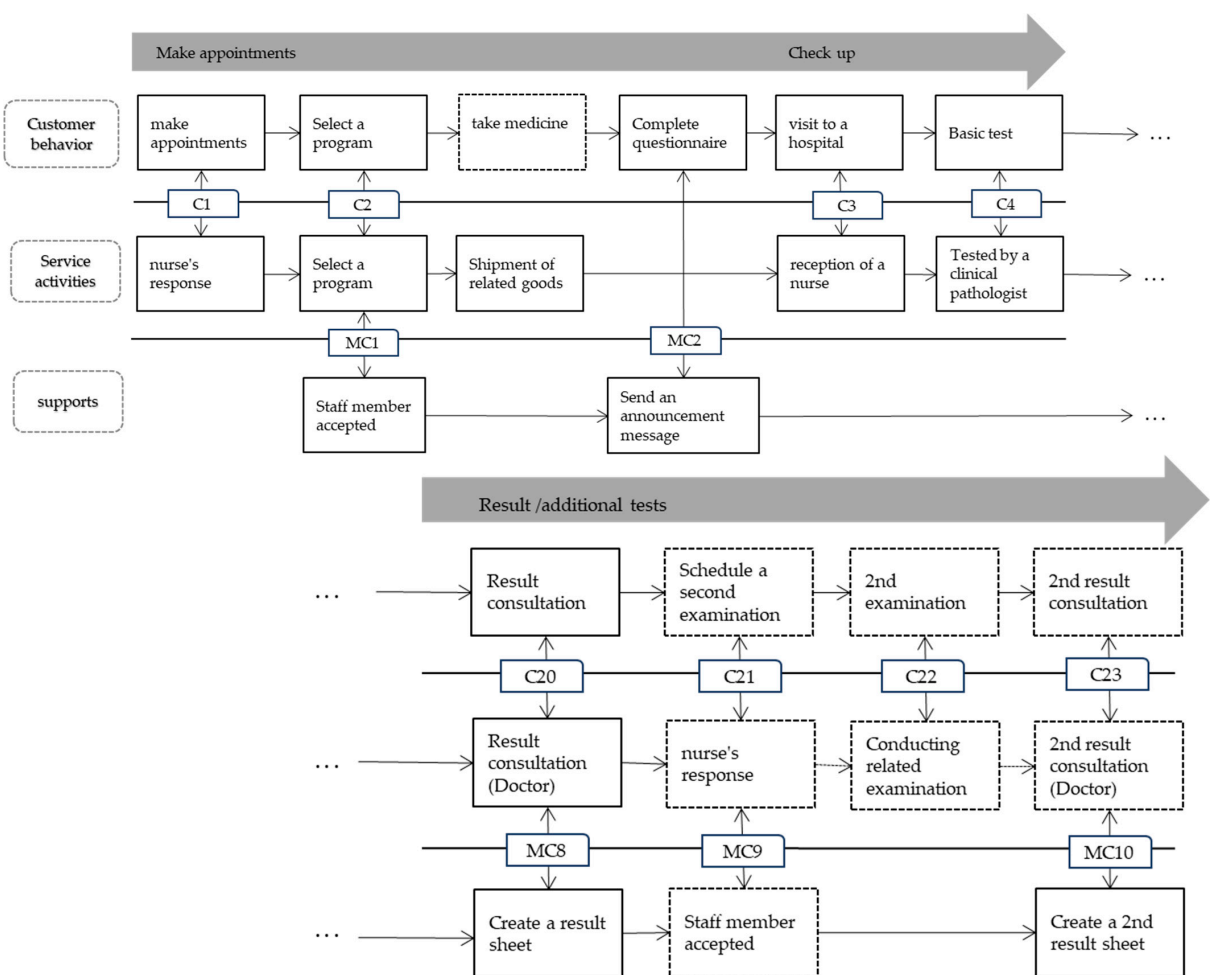
The service blueprint of health examination.

Figure 2 depicts the process of the health examination service for customers to book a medical examination, undergo a checkup, and learn the results after the examination. The service blueprint is read from left to right. The part where the service provider’s actions and physical evidence are linked to the customer, that is, the interaction line, denotes the moment of truth (MOT). The chart’s horizontal axis depicts the actions of customers and service providers in chronological order, whereas the vertical axis distinguishes the various locations of service actions.

Interaction behaviors were classified as visible/invisible to customers and internal interactions. Moreover, arrows connected the flow of service activities related to customer behavior, and dotted lines indicated optional processes.

Patients can make medical checkup appointments online, by phone, or in person at the hospital. They can also choose the appropriate examination program based on the instructions of the responsible staff (nurse). Subsequently, customers fill out the sent questionnaire and receive information from the system before scheduling an appointment, and they are ready for an examination.

Next, patients visit the hospital on the scheduled date, receive guidance from the nurse in charge, and perform various tests following the basic examination by the clinical pathologist. The examination time varies depending on the examination item, and a guardian may be required to accompany the patient. The results are available a few days after the examination, and the patient returns to the hospital to consult with a doctor or nurse. If no specifications or problems exist, the results can be communicated by wire prior to the visit, and the process is immediately terminated. However, if additional tests are required, the second test will be scheduled after reviewing the results. At this time, important clients are assigned the same counseling nurse from the initial appointment, but the majority are assigned at random. If a secondary examination is scheduled, and all additional examinations and results consultations are completed, the medical examination process will be completed, or treatment will be administered if necessary.

The activities shown to the patient in the service blueprint are the nurse’s response to the appointment, the action of giving the checkup list, the guidance of the employee, examinations conducted by the doctor or the clinical pathologist, and so on. Meanwhile, support activities, such as informing employees of the reservation’s contents, sending reservation completion e-mails through the system, and filling out results, are not directly exposed to customers, but they help ensure that services within the line of sight are performed normally. For example, it is a visible service for the patient to explain the results of the examination to the patient and recommend it to be assigned to a related specialist if additional treatment such as cancer is needed, but the staff’s efforts to prepare the accompanying result sheet and the system used to make an appointment with a cancer specialist are support services that are accompanied outside the line of visibility.

The service blueprints included all activities in this manner because the services provided to the customer must be performed as a system, regardless of whether they are visible to the customer. However, these distinctions can serve as the foundation for future analysis. For example, when a service is inefficient, it must be classified to be analyzed, which can determine whether or not it is visible to the customer (behind the line of sight). This modified process can then be constructed accordingly. If the invisible support system needs to be modified, we may notify the customer as soon as possible and then correct it with minimal inconvenience. However, because the customer is already involved in the process, problems seen directly from the customer’s point of contact differ in the actions.

### 3.2. MOT for Health Examination Service

Most process activities have customer contact points, leading to customer dissatisfaction. Service failures can be prevented by analyzing items that may cause customer dissatisfaction at each point of contact. This was examined through in-depth interviews with academic and hospital experts.

When a customer comes in for a checkup, he or she is most likely to make a mistake at C3, where he or she meets with the nurse in charge. It can be interpreted that prior to C3, reservations or questionnaire completion are frequently carried out by representatives such as family members rather than direct contact by older customers. It is also assumed that the response manual, including the older adult, is meticulously prepared. In other words, even if the customer is an older adult, there will be no unsatisfactory factors at this point. However, at C3, which is prone to dissatisfaction, there are numerous factors that may make older adult customers feel uncomfortable when meeting with nurses. These elements are broadly classified as service and equipment. First, let us look at the service section.

Currently, most examination institutions do not require education for older adult service. As a result, the members of the examination institution did not fully meet the needs of the older adult due to a lack of understanding. Moreover, service provider’s kindness or sensitivity toward the older adult may be lacking. Even if you participate in older adult care education, it is difficult to claim that you are highly professional because your doctor is only delivering information to the staff. Unlike online and over the phone, older adults may feel uneasy when visiting a medical examination facility for the first time. At this point, a thoughtful response is required.

It may be difficult to adapt when they visit due to a lack of auxiliary facilities for the older adult, such as old facilities and waiting areas, at the same point of contact.

This is true not only for service providers, but also for the environment in which the service is provided (i.e., facilities). Auxiliary facilities, such as old facilities and waiting areas, are insufficient at the same point of contact, making it difficult to adapt when the older adults visit for examination.

To avoid dissatisfaction at this point of contact, special management personnel must be deployed, service education for the older adult must be implemented, and related manuals must be established. Such dedicated personnel are required to participate in Senior Nursing Society and Senior Nursing Association education. Moreover, if circumstances permit, organizing education on the older adult experience, physical changes, and considerations will be necessary. In addition to services, improvements to facilities, such as inspectors, chairs, and bed height adjustments for the older adult, are required. Furthermore, inspection guides and service information boards must be simple to understand and visible.

Unsatisfactory elements are also very likely to occur at C4, which is C3’s next contact point. Pathologists and doctors, for example, who interact with customers during the examination process, were not well trained for the older adult, and the service process itself may be difficult for them. To avoid this unfamiliarity and dissatisfaction, a service system that is concerned with the older adult must be established. For example, the older adult can be particularly perplexed when the route to the examination room is complicated. Thus, the one-stop system, which begins with vaccinations for the older adult, will address the confusion that leads to dissatisfaction with the service. Furthermore, at this stage, a coordinator for simplifying procedures for the older adult can improve customer experience. This point is also heavily influenced by the facilities and services. Therefore, efforts should be made to improve facilities, such as attaching the examination order with arrow tape to the bottom of the hospital to improve ease of use or installing a call bell in the bathroom or changing room for emergency use. After the examination, C20 and C22 are expected to have similar aspects of noncompliance to the previously mentioned C3 and C4. In particular, in C20, doctors’ or nurses’ explanations of medical examination results frequently result in poor understanding due to linguistic difficulties. In contrast to C3, which entered the health examination system for the first time, a problem with the results may be predicted, thus requiring a service spirit that reduces anxiety.

## 4. Discussion and Conclusions

This study employed service blueprints to delineate the process of health examination services and establish a comprehensive understanding of these services from a systemic perspective. The service blueprint technique is widely used in research to understand the delivery process of intangible services, and it is also commonly applied in studies related to hospital operations [23,24]. This study aims to enhance the level of detail and professionalism by focusing specifically on older adults and their health examination services. It will utilize service blueprints as a framework, while also considering the sequential order of behavior and the interaction between service providers. An in-depth investigation was performed by segmenting and examining services, identifying their respective customer bases, and soliciting expert opinions. This allowed for the identification of potential areas in the service process where customer complaints may arise. Furthermore, the associated service processes were analyzed.

These analysis results directly identify potential mistakes that cause customer dissatisfaction and aid in service improvement. Results reveal a high level of dissatisfaction at the contact point where the actual customer first visited the medical examination institution, and the contact point where he or she went for consultation on the results. The majority of dissatisfaction was caused by service providers’ lack of understanding of the older adult or a lack of facilities. In addition, the physical environment of the facility or service is an important component of service quality and has been analyzed in many ways using, for example, SERVQUAL [25]. Efforts can also be made to improve the convenience of more specialized facilities for the older adult, such as equipment that informs the order of examination, chairs in waiting rooms, and call bells in restrooms.

In contrast, improving a customer’s dissatisfaction situation can lead to customers being impressed and satisfied. In practice, the critical incident methodology identifies and analyzes customer dissatisfaction and satisfaction components to compare their impact on customer satisfaction [26]. In other words, rather than managing negative factors to reduce the likelihood of mistakes, it is critical to lead them to positive experiences. For example, a large hospital has a dedicated counselor who communicates with one customer from start to finish of the service process. Although the person in charge lacks professional knowledge of the older adult, changing to an impressive positive factor is possible simply by having the same employees respond to the C3 and C20 contacts.

Furthermore, other large hospitals that have been in the community for a long time have shown efforts to respond to the older adult (who are their main customers) with consideration, despite the fact that their buildings and institutions are out of date. Securing a dedicated workforce for the older adult and providing senior citizens-specific education to employees who work in areas visible to customers can be excellent alternatives to reduce the possibility of complaints and increase the possibility of satisfaction. In particular, C20 points are places where failure to provide specialized services for older adults can cause great dissatisfaction, but where many positive factors can emerge. It is likely to be a very satisfying point for older adults, who find it difficult to visit multiple hospitals, because they can consult with family medicine specialists after the checkup and take care of them immediately if treatment is required.

It is just as important to manage customers’ critical satisfaction points as it is to manage dissatisfaction factors. Therefore, at the end of the examination, customer satisfaction should be investigated to prepare a system for immediate response to complaints and continuous feedback. Identifying customer dissatisfaction and responding to the point of contact in the service blueprint allow for analysis of which contact point causes dissatisfaction or satisfaction. This analysis will ultimately improve service quality.

As such, this study is significant because it provided basic information on service improvement by allowing service operators to identify areas that may cause customer dissatisfaction or are vulnerable to failure. In particular, this study can provide practical assistance in employee training and service development by schematizing service processes in the chronological order of customer behavior. Moreover, this study defined the service process and analyzed the point at which the mistake occurred from the perspective of the service provider and observer. However, a more robust conclusion could be obtained if the analysis were conducted from the customer’s perspective. If the accumulated data, such as customer centers, can be used to find and analyze the satisfaction/dissatisfaction factors at all customer experience interfaces, more in-depth research will be possible. This is because service characteristics can be analyzed and objective service evaluation indicators can be derived by developing quality customer requirements and conducting additional research on experts and customers’ opinions. Finally, this study analyzes the cases of general hospitals in Korea, so it is difficult to compare them to other countries. In the future, if research is conducted in other countries in the form of the same analysis, more abundant implications will be obtained.

## Figures and Tables

**Table 1:** The overview of the hospitals included in this study.

Hospitals	A	B	C	D
Types of Hospitals	general hospital			
Location (Population of a city)	Busan (3.3 million)	Yeonggwang (0.05 million)	Busan (3.3 million)	Suwon (1.19 million)
Hospital Size (Number of beds)	432	322	378	1161
Operating Revenue	$37 million	$31 million	$15 million	$560 million
Number of Doctors	65	25	83	675
Experts involved in the survey	1 staff nurse 1 Ph.D. in Nursing 2 Ph.D.s in Business 1 Ph.D. in Economics	1 staff nurse 1 Ph.D. in Social Welfare 1 Ph.D. in Public Health 2 Ph.D.s in Business 1 Ph.D. in Economics	1 staff nurse 1 Ph. D. in Nursing 2 Ph. D. s in Business 1 Ph. D. in Economics	1 staff nurse 1 Medical Doctor 2 Ph. D. s in Business 1 Ph. D. in Economics

## Data Availability

Data sharing is not applicable to this article.
